# Multiple Hospital Transfers Among MOQI Nursing Home Residents: The Influence of Race

**DOI:** 10.1093/geroni/igab046.2122

**Published:** 2021-12-17

**Authors:** Kelli Canada, Elizabeth Fritz, Amy Vogelsmeier, Marilyn Rantz, Lori Popejoy

**Affiliations:** 1 University of Missouri, Columbia, Missouri, United States; 2 Sinclair School of Nursing, Columbia, Missouri, United States; 3 University of Missouri - Columbia, Columbia, Missouri, United States

## Abstract

Missouri Quality Initiative (MOQI) was a CMS-funded enhanced care and coordination provider demonstration project (2012-2020) that successfully reduced avoidable hospitalizations and improved nursing home (NH) care quality. Little is known about the influence of race in multiple hospital transfers from NHs. Using a mixed-methods approach we analyzed hospitalization root cause analysis data from 2017-2019 for 1410 residents in 16 MOQI NHs. There were 113 residents who were transferred 609 times. Those with multiple transfers (four or more transfers/year) were compared by race and key characteristics (e.g., code status, diagnosis). A subset of residents with multiple transfers were examined qualitatively to identify and describe key cases. Findings suggest that Black residents have a higher probability for multiple transfers. Findings highlight the need for transfer prevention efforts for Black residents including early assessment and intervention, early/frequent discussion about goals of care, advance directives, resuscitation status, and family/resident understanding of treatment effectiveness.

